# Bringing together raptor collections in Europe for contaminant research and monitoring in relation to chemicals regulations

**DOI:** 10.1007/s11356-017-0096-x

**Published:** 2017-09-16

**Authors:** Paola Movalli, René Dekker, Jan Koschorreck, Gabriele Treu

**Affiliations:** 1 Naturalis Biodiversity Center, Darwinweg 2, 2333 CR Leiden, Netherlands; 2 German Environment Agency (Umweltbundesamt), Bismarckplatz 1, 14193 Berlin, Germany; 3 German Environment Agency (Umweltbundesamt), Fachbereich Chemikaliensicherheit, Fachgebiet IV 2.3 Chemikalien, Wörlitzer Platz 1, 06844 Dessau-Roßlau, Germany

**Keywords:** Raptor, Environmental contamination, Biomonitoring, Natural history museum, Environmental specimen bank, Meta-database, Chemicals legislation

## Abstract

Raptors are good sentinels of environmental contamination and there is good capability for raptor biomonitoring in Europe. Raptor biomonitoring can benefit from natural history museums (NHMs), environmental specimen banks (ESBs) and other collections (e.g. specialist raptor specimen collections). Europe’s NHMs, ESBs and other collections hold large numbers of raptor specimens and samples, covering long periods of time. These collections are potentially a valuable resource for contaminant studies over time and space. There are strong needs to monitor contaminants in the environment to support EU and national chemical management. However, data on raptor specimens in NHMs, ESBs and other collections are dispersed, few are digitised, and they are thus not easy to access. Specimen coverage is patchy in terms of species, space and time. Contaminant research with raptors would be facilitated by creating a framework to link relevant collections, digitising all collections, developing a searchable meta-database covering all existing collections, making them more visible and accessible for contaminant research. This would also help identify gaps in coverage and stimulate specimen collection to fill gaps in support of prioritised contaminant monitoring. Collections can further support raptor biomonitoring by making samples available for analysis on request.

This commentary argues the case for enhanced collaboration and integration between raptor specimen collections in Europe, including natural history museums (NHMs), environmental specimen banks (ESBs) and other collections (e.g. specialist raptor collections), with a view to supporting contaminant research using raptor specimens.

## Raptors are good sentinels of environmental contamination

In part, due to their unique biology, habits and physiology, birds are highly sensitive indicators of local environmental quality, including the presence of contaminants (Backer and Miller 2016). Raptors (birds of prey, owls and scavengers) are especially suitable for monitoring substances that are persistent and bioaccumulate in the environment because (a) they are typically relatively long-lived, apex predators, (b) they effectively integrate contaminant exposure over time and over relatively large spatial areas, (c) they are relatively easily captured which facilitates sampling (of blood, feather, preen gland oil) and/or their eggs can be sampled without sacrificing or harming animals and (d) populations can be relatively easily monitored and quantified (e.g. Furness 1993; Sergio et al. 2005, 2006; Movalli et al. 2008a, 2017). Non-migratory raptor species are of particular value for contaminant exposure monitoring as the potential source of exposure is more easily identified. Movalli et al. (2008b) found that existing national and sub-national monitoring initiatives need to be reinforced and coordination at a pan-European scale improved. However, a recent overview of existing raptor contaminant monitoring activities in Europe (Gómez-Ramírez et al. 2014) reveals widespread capability and expertise. Types of samples that can be used have recently been reviewed and discussed by Espin et al. (2016).

## Europe’s NHMs and ESBs hold large raptor collections, covering long periods of time—allowing for expansion of contaminant studies over space and time

Contaminant research and monitoring with raptors often involves new fieldwork to collect current-day raptor samples from the geographic location of concern as well as control samples from areas thought to be free from contamination. However, this research may also benefit from the use of raptor samples previously collected and stored in NHMs, ESBs and other collections.

NHMs across Europe house large collections of raptor specimens, mostly skins, bones and eggshells, originating from most regions of Europe, dating from the eighteenth century to modern times. While these collections were not designed to serve contaminant monitoring, these specimens can deliver data, particularly as analytical equipment and methods develop, to extend analysis of contaminant exposure over space and time (Campbell and Drevnik 2015). Feathers in particular are known to be a suitable matrix to analyse a wide range of contaminants, though other raptor tissues, when available, may be better matrices for the study of organic compounds (Movalli et al. 2017).

Complementing NHMs, there has been a steep rate of establishment of environmental specimen banks (ESBs) since the first ESB became established in the 1960s (Küster et al. 2015). ESBs collect, process and archive environmental (and sometimes also human) samples in a systematic manner for retrospective chemical analysis. A small number of ESBs hold raptor samples and there is strong interest in others to do so, given a clear link between contaminant monitoring in raptors and regulatory contaminant monitoring needs. ESB raptor specimens tend to be more recent than those held in NHMs, though some NHMs also collect and bank modern day raptor samples. ESBs archive less durable raptor tissues at low or ultra-low temperatures. These include eggs and egg contents (Day et al. 2014), feathers (Garcia-Seoane et al. 2017) and pectoral muscle, liver, kidney, brain, fat, tarsus and feathers (Walker et al. 2014). Such samples are specifically gathered with contaminant monitoring in mind, are amenable to the study of a wider range of contaminants and, like NHM specimens, allow extension of analysis of contaminant exposure over space and time (Rocque and Winker 2005; Odsjö 2006; Day et al., 2014).

## The value of retrospective studies

While older samples from NHMs are only of relevance to the study of very persistent legacy contaminants (e.g. Hg, DDT), more recent samples from ESBs (and some NHMs) can be used to study both legacy contaminants and chemicals of emerging concern (CECs, e.g. neonicotinoides, NSAIDs, second-generation anticoagulant rodenticides, perfluorinated compounds).

Retrospective chemical analysis of archived samples to measure contaminant values in samples that pre-date the widespread use or anthropogenic emissions of the contaminant in question can provide reference values^1^ for exposure to certain contaminants, aiding the interpretation of observed modern-day values. Such retrospective analysis can elucidate the range in natural exposure to certain contaminants and help detect any time-trends in contaminant exposure. It is also possible to contextualise contaminant values from retrospective studies with reference to contemporary ringing data, raptor monitoring data, winter bird census data and long-term climate data (Lister 2011). As analytical techniques such as stable isotope research emerge rapidly (Day et al. 2014), ESBs and NHMs can be utilised as robust and economical sample archive to shed light on so far open research questions.

Retrospective studies of changing exposure to contaminants over time can have impact on chemical management and raptor population health. For example, Berg et al. (1966) found a 10- to 20-fold increase in feather mercury among raptors and seed-eaters after the introduction of alkylmercury seed dressings (fungicides) in Europe in the 1940s, prompting the banning of such dressings and a consequent decline in mercury concentrations in feathers (Westermark et al. 1975). Ratcliffe (1967) and Hickey and Anderson (1968) demonstrated eggshell thinning in raptors following introduction of DDT in 1947, using museum specimens, contributing with other studies (see Kiff 2005) to the eventual ban of DDT in many countries (Rocque and Winker 2005). Similarly, in the 1960s, Jensen et al. (1969) identified PCBs in samples from white-tailed sea eagles in Sweden and traced PCB contamination back to the 1940s. Recently, the NSAID diclofenac was identified as being responsible for the decline of certain vulture populations in Asia and led to marketing restrictions of the substances (Oaks et al. 2004).

## Relevance to international and regional chemical regulation

Monitoring of contaminants in raptors, drawing on specimens from NHMs, ESBs and other collections, together with current-day sampling by research projects, is of relevance to chemical management including identification of CECs, early warning, hazard and risk assessment during substance evaluation, monitoring of wildlife exposure, post-marketing vigilance and effectiveness evaluation of legal measures. International clients of monitoring data are e.g. the Stockholm Convention for persistent organic pollutants (POPs) and the Minamata Convention (UNEP 2013), which address the exposure of the environment to chemicals and their food web accumulation. In Europe, there are several legal frameworks involving chemical monitoring data, including those for veterinary medicines (EC 2001), industrial chemicals (REACH, EC 2006), plant protection products (EC 2009) and biocides (EC 2012). Top predators including raptors are among the protection goals under the EU Water Framework Directive (EC 2000), OSPAR Convention (OSPAR 2007), EU Marine Strategy Framework Directive (EC 2008) and Helsinki Convention (HELCOM 2014). More specifically, raptor monitoring data can be supportively used in a weight of evidence approach by national competent authorities to clarify the initial concern during substance evaluation and to decide on the most appropriate follow-up to address the concern.

While only 22 substances of the > 9000 registered under REACH are identified as PBT (persistent, bioaccumulative and toxic) substances and/or vPvB (very persistent, very bioaccumulative) substances, the estimated number of PBT/vPvB chemicals may be higher (Strempel et al. 2012). Raptor monitoring data, involving the use of specimens from NHMs and ESBs, could usefully throw light on the exposure situation including the bioaccumulation of these substances.

Contaminant monitoring in raptors, involving retrospective studies supported by NHMs, ESBs and other collections, can be a powerful complement to the HBM4EU human biomonitoring project,^2^ with a view to providing early warning of emerging contaminant problems, assessing the effectiveness of EU chemicals regulation and protecting human and environmental health (Kolossa-Gehring et al. 2016).

## Limits of the current situation

At present, the use of raptor collections in NHMs, ESBs for contaminant monitoring is constrained by a number of factors relating to the visibility and accessibility of these collections. The various collections are dispersed around Europe and there is no central database of specimens that researchers can consult in order to inform the design and prioritisation of research and monitoring programmes. The creation of a European meta-database of specimens is hampered by the fact that few raptor collections are digitised, though there is strong interest to achieve this. And while initiatives such as Scientific Collections International (www.scicoll.org) and the Global Biodiversity Information Facility (GBIF) link NHMs and the International ESB Group (www.inter-esb.org) links ESBs, there is no network linking both NHMs and ESBs (and other relevant collections) for the purpose of making raptor specimens more visible and accessible for chemical monitoring (and other) purposes.

## The solution—a framework linking NHM, ESB and other raptor collections

We suggest that contaminant research and monitoring with raptors, in support of EU and regional chemicals law and policy, would be greatly facilitated by creating a framework to link relevant collections. This would involve digitising all relevant collections and developing a searchable meta-database of these collections (and of any related existing contaminant data), making them more visible and accessible. This idea has strong support from a wide range of NHMs and ESBs in Europe. Furthermore, archived raptor samples should be made available on request by external collaborators to allow for high impact research.

Construction of the meta-database would involve developing standards for the digital description and online publication of raptor specimens and related data, and developing systems and protocols for the web-based exchange, analysis and visualisation (mapping) of this data. Such a meta-database would enable a clear overview of the temporal and geographic extent (per species) of existing collections. This would in turn (a) stimulate and inform the design of contaminant research using raptors; (b) help to identify gaps in coverage and stimulate greater collection of contemporary specimens to fill gaps in support of prioritised contaminant monitoring. Few ESBs currently collect raptor specimens but many have shown interest in doing so given the relevance to EU regulations. New regional collection centres may also be established to help fill geographical gaps and promote harmonisation of methods. Such a meta-database would create a European resource bringing together NHMs and ESBs and other collections in support of contaminant monitoring with raptors, thereby offering a new way to make use of these collections. The meta-database would complement the existing GRSciColl database of museum specimens and could be linked to the IpChem database (https://ipchem.jrc.ec.europa.eu), which brings together chemical exposure data on environmental specimens and human populations for contaminant research and monitoring in Europe.

## Footnotes

1 A reference value is the concentration of an element or compound in a given tissue/matrix at a fixed point in time in the past and at a specified location.

2 see: www.eea.europa.eu/themes/human/human-biomonitoring
